# Cephalosporin-resistant Pneumococci and Sickle Cell Disease

**DOI:** 10.3201/eid1108.050152

**Published:** 2005-08

**Authors:** Martha L. Miller, Caroline A. Obert, Geli Gao, Najat C. Daw, Patricia Flynn, Elaine Tuomanen

**Affiliations:** * St. Jude Children's Research Hospital, Memphis, Tennessee, USA

**Keywords:** infectious diseases, Antibiotic resistance, multi-drug resistance, anemia, sickle cell

## Abstract

Sickle cell anemia patients have 600 times the risk for invasive pneumococcal disease than their healthy peers. High-level cephalosporin resistance was described in the 1990s in healthy children from Tennessee, but its prevalence in sickle cell disease patients is unknown. Pneumococcal isolates from sickle cell disease patients from Tennessee were subjected to multilocus sequence typing to characterize antimicrobial drug–resistant strains. Twenty-one percent of strains were resistant to cefotaxime and penicillin. Of the 14 cephalosporin-resistant strains, 9 were sequence types previously described as highly cephalosporin resistant, while resistance was found for the first time in 3 clones: Maryland^6B^, ST660, and a novel clone, ST1753. High-level cephalosporin resistance exists in more settings than initially recognized, and its high prevalence in sickle cell disease patients may decrease the efficacy of third-generation cephalosporins in invasive pneumococcal disease.

## Abstract 2

Increasingly resistant bacteria in sickle cell disease patients indicate need to evaluate extendedspectrum cephalosporin therapy.

*Streptococcus pneumoniae* is a gram-positive bacterium that causes substantial illness and death in children. Children with sickle cell disease have an increased risk for invasive infection from this pathogen. Before the routine use of prophylactic measures, invasive pneumococcal disease was 600 times more likely to develop in patients with sickle cell disease than in their healthy peers (1). Thus, colonization with pneumococci is viewed as a high-risk event for sickle cell disease patients.

The risk for fatal infection increases if the patient is colonized with antimicrobial drug–resistant pneumococci. The prevalence of colonization with pneumococci is generally the same in healthy persons (12%) and sickle cell disease patients (7%) (2). However, penicillin-resistant pneumococci are consistently more common in children with sickle cell disease (62% versus 41% in healthy children) (2). A similarly high incidence of penicillin resistance (55%) in pneumococci infecting sickle cell disease patients was reported by Daw et al. (3) and has been sustained throughout the 1990s (4).

In the early 1990s, the Centers for Disease Control and Prevention described a series of community-acquired invasive infections in healthy children from Memphis, Tennessee, caused by pneumococci with unusually high resistance to extended-spectrum cephalosporins > (5). These strains displayed MICs of cefotaxime and ceftriaxone from 4 to 32 μg/mL, exceeding the MICs of penicillin by as much as 5-fold (6). This finding is of clinical importance since it precludes using cephalosporins as a treatment option (6,7). Richter et al. extended the analysis of this resistant Tennessee cluster and identified a novel clone termed TN^23F^-4 with MIC values of third-generation cephalosporins as high as 32 μg/mL (8,9).

The epidemiology of the cephalosporin-resistant TN^23F^-4 clone in sickle cell disease patients is unknown. Colonization with this clone would have implications in terms of drug therapy, since this patient population routinely receives standard doses of extended-spectrum cephalosporins to treat invasive pneumococcal disease. Infection with a strain exhibiting high-level cephalosporin-resistance could result in treatment failure. In this study, we reexamined pneumococci collected from sickle cell disease patients in Memphis, Tennessee, from 1994 to 1995, the time of the original description of the TN^23F^-4 clone, to determine the prevalence of this clone and any other highly cephalosporin-resistant clones circulating in the sickle cell disease population.

## Materials and Methods

### Pneumococcal Strains

Sixty-four nasopharyngeal isolates were collected from 42 patients between July 1994 and December 1995 at the Mid-South Sickle Cell Center (3). Frozen strains were recovered by overnight growth on blood agar plates at 37°C, followed by resuspension in 15% glycerol solution. Strains were refrozen at –80°C for further use. Since this study was retrospective and used clinical pneumococcal strains, institutional review board permission was granted to review characteristics specific to the isolates themselves. Limited patient demographics were obtained, including patient diagnosis and penicillin prophylaxis. Antimicrobial susceptibility testing was conducted as described (3). Susceptibility breakpoints were defined according to the Clinical and Laboratory Standards Institute (formerly NCCLS) guidelines for the years 1994 and 1995 (10,11). Pneumococcal isolates were serotyped by the slide agglutination method (12) with the Pneumotest-Latex Kit (Statens Serum Institut, Copenhagen, Denmark).

### Genomic DNA Preparation

Genomic DNA was prepared by sodium dodecyl sulfate (SDS) lysis (13) and standard phenol:chloroform extraction (14). Each strain was grown in 20 mL casein/yeast broth (15) at 37°C in 5% CO_2_ until turbid. Bacteria were harvested by centrifugation; resuspended in 500 μL (1:20 volume) iced buffer containing Tris-HCl, glucose, and EDTA (13); and treated with 15 μL 10% deoxycholate and 1.25 μL 10% SDS. After incubation at 37°C for 30 min, 30 μL 10% SDS was added and gently mixed by inversion. The mixture was incubated with 200 μg/mL proteinase K (Invitrogen, Carlsbad, CA, USA) overnight. An equal volume of phenol:chloroform:isoamyl (Invitrogen) was added to each sample and centrifuged for 5 min at 12,000 rpm. The upper phase was treated 2 more times. The extract was treated with 10% volume of 3 mol/L sodium acetate. DNA was then precipitated by adding 2 volumes of cold 95%–100% ethanol. The DNA pellet was treated twice with cold 70% alcohol. The resultant sample was air dried and resuspended in 20 μL distilled water.

### Multilocus Sequence Typing

To assign the strains to a sequence type (ST), 7 housekeeping genes were subjected to polymerase chain reaction (PCR) amplification and DNA nucleotide sequencing: *aroE* (shikimate dehydrogenase), *gdh* (glucose 6-phosphate dehydrogenase), *gki* (glucose kinase), *recP* (transketolase), *spi* (signal peptidase I), *xpt* (xanthine phosphoribosyltransferase), and *ddl* (D-alanine-D-alanine ligase). The primer sets were obtained from the multilocus sequence typing (MLST) Web site (http://www.mlst.net). Fifty-microliter reaction mixtures were prepared with 1.25 U Taq polymerase (Applied Biosystems, Foster City, CA, USA), 1× Taq polymerase buffer (10 mmol/L Tris-HCl [pH 8.3], 50 mmol/L KCl), 1.5 mmol/L MgCl, 0.2 mmol/L each deoxynucleoside triphosphate, and 0.2 mmol/L each primer. One microliter of genomic DNA was added to each reaction. The following parameters were used for amplification: denaturation at 95°C for 5 min, 30 subsequent cycles of amplification, each consisting of 1 min at 95°C, 1 min at 50°C, and 30 s at 72°C, with a final extension at 72°C for 7 min. PCR products were analyzed by electrophoresis on a 1.0% wt/vol agarose gel, and the amplicon size was evaluated by comparing it with 1-kb ladder (Invitrogen). PCR products were purified by using the QIAquick PCR purification kit (Qiagen, Valencia, CA, USA). Sequencing, with forward and reverse primers, was performed on the ABI 377 DNA sequencer with Big Dye chemistry (Applied Biosystems), according to ABI protocols, by the St. Jude Hartwell Center for Bioinformatics and Biotechnology.

### Phylogenetic Analysis

Sequencing results were assembled by using SeqAssem version 09/2004 (http://www.gwdg.de/~dhepper/Sequences were subsequently queried against the NCBI nonredundant database by using both nucleotide-BLAST [blastn] and protein-BLAST [blastp] programs and compared by alignment with the ClustalW algorithm in BioEdit (16). Concatenated DNA alignments of the 7 housekeeping genes were used for phylogenetic analysis. The phylogenetic relationship among the 64 pneumococcal strains was inferred by using the Bayesian approach (17), a variant of the maximum likelihood algorithm. Although eBURST (18,19) defines clonality based on 6/7 shared alleles, the Bayesian approach allows resolution of clonality based on 7/7 alleles. Thus, the Bayesian approach allows branch placement, due to differing alleles, into a paraphyletic clade versus clustering as a monophyletic clade. Clade credibility for the consensus tree topology was calculated by using MrBayes version 3.0b4 (20) with the following parameters: 1 million generations, 4 simultaneous Monte Carlo chains, and exclusion of the first 1,000 trees. The tree was rooted by using data from the *S. pneumonia*e TIGR4 strain (21) as an outgroup. An evolutionary model of nucleotide substitution was selected by using the MrModeltest program version 2.1 (22).

## Results

We analyzed 64 nasopharyngeal strains from patients in whom homozygous sickle cell disease (HgbSS), hemoglobin SC sickle cell disease (HgbSC), or hemoglobin Sβ^+^ thalassemia (HgbSβ^+^) was diagnosed. Antimicrobial drug–susceptibility results are represented in Tables 1 and 2. Fifty-one percent (33/64) of the strains were penicillin-resistant (intermediate strains included). Of these strains, 14 (42%) of 33 were resistant to cefotaxime. No serotypes consistently correlated with antimicrobial drug resistance (Table 2).

**Table 1:** Distribution of β-lactam resistance in nasopharyngeal pneumococcal isolates from sickle cell disease patients

Penicillin susceptibility*	No. isolates	Cefotaxime susceptibility*	No. isolates
Sensitive	31	Sensitive	31
Intermediate	24	Sensitive	19
		Intermediate	3
		Resistant	2
Resistant	9	Intermediate	3
		Resistant	6

*Definition of susceptibility per Clinical and Laboratory Standards Institute (formerly NCCLS) guidelines from 1994 and 1995 (10,11). Sensitive, penicillin V MIC<0.06 μg/mL, cefotaxime MIC<0.05 μg/mL; intermediate, penicillin V MIC 0.12–1 μg/mL, cefotaxime MIC 1 μg/mL; resistant, penicillin or cefotaxime MIC>2 μg/mL.

**Table 2:** Multilocus sequence types of penicillin- and cephalosporin-resistant isolates*

Strain	Serotype	Allele numbers							Sequence type	Resistance		MIC values	
		*aroE*	*gdh*	*gki*	*recP*	*spi*	*xpt*	*ddl*		PNV	CTX	PNV	CTX
38	3	8	37	9	29	2	12	NA	Novel	I	S	0.38	0.50
3	3	7	15	2	10	6	1	22	180	S			
52	4	10	5	4	5	13	10	18	205	S			
46	4	10	5	4	5	13	10	18	205	S			
61	4	16	13	4	4	6	10	18	899	S			
48	6	8	37	9	29	2	12	53	344	I	S	0.38	0.19
1	6	7	6	9	2	6	1	67	384	I	I	1.00	0.75
49	6	5	7	4	10	10	1	27	460	I	NA	0.38	
63	6	5	7	4	10	10	1	27	460	I	S	0.38	0.50
35	6	1	25	1	8	15	20	14	660	I	I	1.00	0.75
81	6	1	25	72	1	15	20	28	690	I	S	0.094	0.094
84	6	2	13	8	6	6	3	NA	1754	I	NA	0.25	
75	6	7	25	4	4	15	20	NA	Novel	I	S	0.094	0.094
13	6	1	5	4	5	5	3	101	NT3	I	S	0.38	0.19
29	6	1	5	4	5	5	3	101	NT3	I	S	0.94	0.25
19	6	7	6	9	2	6	1	67	384	R	R	3.00	3.00
33	6	7	6	1	2	6	15	14	146	S			
37	6	7	6	1	2	6	15	14	146	S			
40	6	7	6	1	2	6	15	14	146	S			
45	6	7	6	1	2	6	15	14	146	S			
39	6	7	13	8	6	10	6	14	176	S			
76	6	7	15	2	10	6	1	22	180	S			
44	6	1	5	1	5	1	1	8	425	S			
85	6	7	25	4	4	15	20	1	647	S			
30	6	1	25	72	1	15	20	28	690	S			
43	6	1	25	72	1	15	20	28	690	S			
31	6	7	61	1	1	17	1	NA	1752	S			
4	11	2	5	29	12	16	3	14	62	S			
20	11	2	5	29	12	16	3	14	62	S			
22	14	2	8	7	4	6	1	1	67	R	I	4.00	1.00
27	14	2	8	7	4	6	1	1	67	R	I	4.00	1.00
57	14	2	8	7	4	6	1	1	67	R	I	2.00	1.00
59	14	2	8	7	4	6	1	1	67	R	I	3.00	1.50
74	14	1	5	4	5	5	27	8	13	S			
56	14	7	5	1	8	14	11	14	124	S			
83	14	7	5	1	8	14	11	14	124	S			
28	15	8	6	14	4	17	4	14	1755	S			
69	15	8	13	1	4	17	4	14	1757	S			
71	15	8	13	1	4	17	4	14	1757	S			
60	19	8	13	14	4	17	4	14	199	I	S	0.125	0.064
70	19	8	13	14	4	17	4	14	199	I	S	0.190	0.094
79	19	8	13	14	4	17	4	14	199	I	S	0.125	0.064
80	19	8	13	14	4	17	4	14	199	I	S	0.094	0.064
24	19	15	16	19	15	6	20	26	236	I	S	0.25	0.25
34	19	15	16	19	15	6	20	26	236	I	S	0.25	0.25
65	19	15	16	19	15	6	20	26	236	I	I	1.00	0.75
42	19	8	20	14	4	17	4	14	1756	I	S	0.190	0.094
77	19	8	20	14	4	17	4	14	1756	I	S	0.125	0.094
78	19	8	20	14	4	17	4	14	1756	I	S	0.190	0.064
41	19	15	NA	NA	NA	6	NA	NA	Novel	I	S	0.25	0.19
32	19	1	10	4	1	9	3	8	43	S			
15	19	8	13	14	4	6	4	14	876	S			
68	22	1	1	4	1	18	58	17	433	S			
2	23	1	8	6	2	6	4	6	37	I	R	1.00	1.50
53	23	1	8	6	2	6	4	6	37	I	R	0.125	1.50
26	23	1	8	6	2	6	4	6	37	R	R	4.00	6.00
47	23	1	8	6	2	6	4	6	37	R	R	8.00	8.00
62	23	1	8	6	2	6	4	6	37	R	R	3.00	8.00
58	23	1	8	6	2	6	4	NA	1753	R	R	3.00	8.00
86	23	1	8	9	2	6	4	6	439	S			
14	23	13	8	9	2	6	4	6	1499	S			
55	ND	29	33	19	1	36	22	31	447	S			
66	ND	5	5	6	1	9	10	14	547	S			
25	ND	7	9	NA	1	14	48	14	Novel	S			

*PNV, penicillin V; CTX, cefotaxime; S, susceptible; I, intermediate; R, resistant; NT, nontypeable; NA, not available; ND, not done.

All strains were subjected to MLST and phylogenetic analysis (Table 2, Figure). Isolates were designated as novel if they possessed ≥1 unrecognized alleles based upon known sequences listed in the MLST database. Isolates were designated as nontypeable (NT) if the allele profile was not listed in the MLST database. The 31 penicillin-sensitive strains were distributed broadly through 19 known STs (13, 43, 62, 124, 146, 176, 180, 205, 208, 425, 433, 439, 447, 547, 647, 690, 876, 899, and 1499), 2 putative novel strains (1752 and pending ST), and 3 NT strains (1755 and 1757). Intermediate penicillin resistance (n = 24) was also broadly represented by 8 known STs (37, 199, 236, 344, 384, 460, 660, and 690), 4 novel STs (1754 and 3 distinct STs with pending designations), and 2 NT sequence types (1756 and pending ST referred to as NT3) (Table 2).

**Figure:**
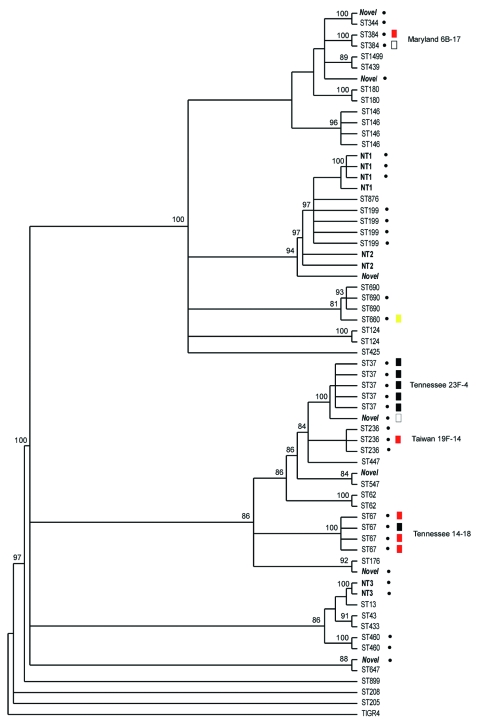
Bayesian analysis of the phylogenetic relationship among pneumococcal isolates from sickle-cell disease patients as determined by multilocus sequence type (MLST). ST, sequence type, as defined by the MLST database. Strains in **boldface** had recognized allele numbers but not recognized profiles according to the current database (listed as NT). Putative novel sequence types (those with unrecognized alleles) are in **boldface** and *italics*. Dots indicate resistance to penicillin. Boxes indicate sequence types determined to be resistant to cephalosporins. Red box, intermediate resistance, previously described; black box, highly resistant, previously described; yellow box, intermediate resistance, not previously described; white box, highly resistant, not previously described. Clade credibility scores >80% are listed on the tree.

In contrast to the broad ST distribution of sensitive and intermediate penicillin-resistant strains, high-level penicillin and cephalosporin resistance was restricted to 4 STs (37, 67, 384, and the novel 1753). Two of these STs have been reported previously to be highly cephalosporin resistant: TN^23F^-4 (ST37) and TN^14^-18 (ST67) (23). Five (8%) of 64 strains were classified as the TN^23F^-4 clone, all of which were highly resistant, and 4 (6%) were designated as TN^14^-18; 1 of these was highly resistant and 3 were intermediate. Two STs, Maryland^6B^-17 (ST384) and Taiwan^19F^-14 (ST236), have been reported to be intermediately cephalosporin resistant (24,25). In this study cohort, 1 Maryland^6B^-17 was highly resistant and 1 intermediate. One of the 3 Taiwan^19F^-14 strains was intermediately resistant, while the other 2 were sensitive. Seven isolates (11%) of the study cohort represented distinct putative novel clones (ST1752, 1753, and 1754; additional designations from MLST database are still pending); 1 of these isolates (ST1753) was highly cephalosporin resistant. ST1753 is closely related to TN^23F^-4 (ST37). Two of the newly recognized cephalosporin-resistant clones (ST384 and 660) were not closely related to each other or the TN clones (Figure).

## Discussion

Although the incidence of carriage of penicillin-nonsusceptible pneumococcus is highly variable, depending on area (from <5% to <50%) (7,26), the incidence is generally increasing worldwide. Children with sickle cell disease routinely receive penicillin prophylaxis as well as empiric therapy with third-generation cephalosporins and have a higher rate of carriage of penicillin-resistant strains (2–4).

We analyzed cephalosporin resistance in pneumococcal nasopharyngeal isolates collected from 1994 to 1995 from patients with sickle cell disease in Memphis, a time and place corresponding to the initial description of the highly cephalosporin-resistant TN^23F^-4 clone. Fifty-one percent of strains were penicillin resistant, a percentage consistent with that seen in many previous sickle cell disease studies (2–4). Strikingly, 14 (21%) of 64 isolates were resistant to cefotaxime, 12.5% at high level, and all of the cephalosporin-resistant strains were also resistant to penicillin. Although this sample is small and precludes the ability for direct comparison, this percentage is much greater than the 4.6% reported for clone TN^23F^-4 (8). Nine of the 14 cephalosporin-resistant strains were either the TN^23F^-4 clone or TN^14^-18, the 2 sequence types reported previously to be highly cephalosporin resistant (5,6,23). Of the remaining 5 cephalosporin-resistant strains, 4 STs, Maryland^6B^-17 (25), Taiwan^19F^-14 (24), ST660, and a novel clone ST1753, had increased levels of cephalosporin resistance not previously described. Of these newly described resistant strains, only the novel clone was closely related to either TN clones, suggesting that high-level third-generation cephalosporin resistance exists in a wider array of backgrounds than previously recognized.

Fifty-three (80%) of the 66 strains had serotypes contained in the Prevnar vaccine (Wyeth Pharmaceuticals, Philadelphia, PA, USA), including all cefotaxime-resistant strains. Routine administration of Prevnar since 2000 (5 years after these strains were collected) is likely to have protected most of the sickle cell disease population from risk for cephalosporin-resistant disease. However, the possibility of colonization with nonvaccine serotypes or the extension of cefotaxime resistance into previously unrecognized backgrounds should not be underestimated.

Aggressive management is warranted to prevent death from invasive pneumococcal infections in children with sickle cell disease. With the increasing prevalence of penicillin and extended-spectrum cephalosporin resistance in the sickle cell disease population, alternative antimicrobial drug therapies may be needed for prophylaxis. If the high prevalence of third-generation cephalosporin resistance is documented in the sickle cell disease population in other geographic areas, extended-spectrum cephalosporins should be reconsidered as empiric therapy when invasive pneumococcal disease is suspected. As antimicrobial drug alternatives are eliminated, vaccination becomes more important as the mainstay of prophylactic management of disease.
